# Feasibility of an online and a face-to-face version of a self-management program for young adults with a rheumatic disease: experiences of young adults and peer leaders

**DOI:** 10.1186/1546-0096-12-10

**Published:** 2014-03-25

**Authors:** Judy Ammerlaan, Harmieke van Os-Medendorp, Lieske Scholtus, André de Vos, Matthijs Zwier, Hans Bijlsma, Aike A Kruize

**Affiliations:** 1 Department of Rheumatology & Clinical Immunology, University Medical Center Utrecht, Utrecht, Netherlands; 2 Department of Dermatology and Allergology, University Medical Center Utrecht, Utrecht, Netherlands; 3 Dutch Rheumatism Patient League (Dutch Arthritis Association), Amsterdam, Netherlands; 4 CBO (TNO Company), Dutch Institute for Healthcare Improvement, Utrecht, Netherlands

**Keywords:** Young adults, Juvenile idiopathic arthritis, Peer leaders, Self-management, e-Health application, Face-to-face training, Feasibility

## Abstract

**Background:**

Based on the self-efficacy theory, an online and a face-to-face self-management programs ‘Challenge your Arthritis’ for young adults with a rheumatic disease have recently been developed. These two courses are led by young peer leaders. The objective of this study was to test the feasibility of the online and face-to-face self-management program.

**Methods:**

Feasibility was evaluated on items of perceived usefulness, perceived ease of use, user-acceptance, and adherence to both programs in young adults and peer leaders. Additional analyses of interactions on the e-Health applications, discussion board and chat board, were conducted.

**Results:**

Twenty-two young adults with a diagnosed rheumatic disease participated in the study: 12 young adults followed the online program and 10 followed the face-to-face program. Both programs appeared to be feasible, especially in dealing with problems in daily life, and the participants indicated the time investment as ‘worthwhile’. In using the online program, no technical problems occurred. Participants found the program easy to use, user friendly, and liked the ‘look and feel’ of the program.

**Conclusions:**

Both the online and the face-to-face versions of a self-management program. ‘Challenge your arthritis’ were found to be feasible and well appreciated by young adults with a rheumatic disease. Because these programs are likely to be a practical aid to health practices, a randomized controlled study to investigate the effects on patient outcomes is planned.

## Background

Numerous rheumatic diseases such as arthritis, fibromyalgia and systemic lupus erythematosus affect children [1]. These diseases and their treatments put extensive demands on children, young adults and their parents, by precisely scheduling daily medication, regular physical exercises, blood tests, and regular visits to a primary care provider or a rheumatologist [2,3]. Many of the young adults with a rheumatic disease experience problems into their adult years [2], as they often have significant disabilities and may require on-going medical treatment [4]. Yet, as all young adults, they have to develop their own identity and independence. As they become more independent, they will gradually have to take over the roles of their parents and become more responsible for their own illness and treatment and thereby become a self-manager. In the same period of this change, they also make the transition from child care to adult care systems. Often they are quite unprepared for and inexperienced in assuming all these adult roles especially since they may not had anything like a normal childhood and adolescence [5-7].

To educate and counsel young adults, we first developed an outpatient transition clinic, a digital portal and an information website [8]. Users of these e-Health applications and young adults from the outpatient transition clinic expressed their needs for a training program in which they could practice self-management skills. To meet these needs, we developed two versions of a self management program ‘Challenge your Arthritis’, based on the Arthritis Self Management Program of Stanford University [9]. The aim of this study was to examine the feasibility of the online and the face-to-face version for young adults and peer leaders with respect to perceived usefulness, perceived ease of use and user acceptance in order to improve and adjust the program if necessary and to implement both versions of the program into daily practice.

## Methods

### Study design

Ethical approval was obtained by the ethics committee of University Medical Center Utrecht. The registration number is 10–325.

We developed an online version, using a safe website and a face-to-face version so young patients could choose to participate in the way that best suited their current lifestyle. All these initiatives were taken in close cooperation with the Dutch Rheumatism Patient League (DRPL) and young adults from the outpatient transition clinic of University Medical Center. Both groups participated actively in the development of the program. For the development, the transition outpatient clinic received a grant from the Dutch Arthritis Association.

The ‘Challenge your Arthritis’ program is a multi-component, interactive program of education, self-management strategies and social support. The aim of ‘Challenge your Arthritis’ is to enhance patients’ self-management in coping with their chronic disease. The program is given by peer leaders. To our knowledge, this is the first self-management program for this specific young group given by peer leaders in the Netherlands.

We conducted this quantitative feasibility study to evaluate perceived usefulness, perceived ease of use and user acceptance of the online or face-to-face version of ‘Challenge your Arthritis’ in participating young adults and peer leaders. Additional qualitative analyses of the digital communication within the online version on chat and discussion board were carried out.

### Participants

Young adults aged 16–25 years, who had registered for the self-management program ‘Challenge your arthritis’ on the website http://www.reumauitgedaagd.nl in January 2011, were asked to participate. Information about the program was spread by placing leaflets in the waiting room of University Medical Centers in the Netherlands and on the website of the Dutch Rheumatism Patient League.

Young adults chose voluntarily either for the online or the face-to-face program. Those young adults who initially did not want to participate in the study were given the opportunity to join the program later on. Inclusion criteria were:

1) Each participant had to have a rheumatic diagnosis made by paediatrician or rheumatologist;

2) Access to a home-based computer with Internet;

3) Each had to be be able to read and write in Dutch; and

4) No previously participation in a self-management program.

Informed consent was obtained from the participant and if young adults were younger then 18 years old, also from their parents. The peer leaders who participated in this study were young adults in the age of 20–30 years who had a rheumatic disease themselves.

### The self-management program

The online and face-to-face version ‘Challenge your arthritis’ were based on self-efficacy theory [10]. Self-management may be enhanced by increasing self-efficacy through practicing, observing others (modeling), exposure to beliefs of others, and by interpretation of physiological and emotional status. All these elements were integrated into ‘Challenge your Arthritis’. From the start of the development of this tool, young, non-professional adults played a central role. In interactive workshops, organized by the multidisciplinary team of the transition outpatient clinic, the young adults decided, together with the professionals, the content, style and format of the online and face to-face training programs.

Next the participants of the program were allowed to use information presented on the website http://www.jong-en-reuma.nl (in Dutch). This website contains information about medical issues and themes like dealing with the consequences of having a rheumatic disease, feeling depressed, exercises, work, study, relationship and intimacy.

The online program consists of password-protected, interactive web-based self-management instruction with three e-Health applications including a chat section, home exercises and a discussion board. Once a week, the group (six participants, two trainers) had a planned group chat for a maximum of 90 minutes. Within the chat, the weekly theme was clarified, goals were set and the participants were allowed to practice, ask questions, play a game or watch a real live story video, based on the weekly theme. The home exercises were discussed and evaluated. After the chat, participants were allowed to work through the program at any time at home and do the exercises (one hour a week). In addition, a discussion board was used by trainers and participants to offer encouragement and share tips.

For six weeks, participants worked chronologically through the program using six themes (Table 1). On average, the total time investment for the participants was 12 hours in a 6-week-period.

**Table 1:** Themes of the ‘challenge your arthritis’ self-management program

**Theme**	**Examples of sub themes**
**1. Are you a self-manager?**	- Introduce yourself, get in touch with the program and the group
	- Goal setting, action planning
**2. Communication**	- Communication strategy
	- Giving and receiving feedback
**3. Feeling blue**	- Pain, fatigue, feeling blue
	- Asking and giving help
**4. Sport and exercise**	- Being active
	- Maintain your plans
	- Healthy nutrition
**5. Relations and intimacy**	- Having a relationship
	- Having sex
**6. Having control over your live and arthritis**	- Evaluation of the personal goals
	- Being responsible and make choices

The face-to-face version of the program was organized during a weekend in a holiday resort. In three days, 12 hours in total, participants (8–12 young adults) and two peer leaders worked through the same program and themes (Table 1) in a similar way as in the online edition. Here also personal goals were set and objectives were clarified.

The ‘Challenge your Arthritis’ program was administered by peer leaders, young adults in the age range of 20–30 years old who also had an arthritis disease. The peer leaders were recruited through the websites of the DRPL, the arthritis youth network (Youth-R-well.com) and the website of the Dutch Arthritis Association. Peer leaders were selected through assessments and interviews conducted by the DRPL. The selection process utilized questions on age, disease duration, motivation, perceptions on self-management and strategies. Finally, the peer leader was trained through a train-the-trainer educational program by the UMC Utrecht and a professional coaching organization (Work21.nl). This program consisted of following the training as participant, knowledge of the different themes, and teaching training skills. The peer leaders had a volunteer contract and received a stipend from the Dutch Arthritis Association for each program worked.

Two moderators assisted the peer leaders during the online and face-to-face versions of the program, but were not ‘visible’ to the participants. These moderators were a professional coach of the train-the-trainer program, transition coordinator, or a communication advisor of UMC Utrecht.

### Variables

#### Demographic variables and internet-related skills

We collected demographic variables including age, sex, diagnosis, disease duration, current treatment, and educational level at baseline by means of an online questionnaire as well as data about the use of Internet and self-reported Internet skills by participants of the online program.

### Primary outcome measure: feasibility of the program

#### Participants

Three months after starting the online version and two weeks after completing the face-to-face version, feasibility was evaluated using the Technology Acceptance Model (TAM) [11,12]. TAM is a prediction and explanation model of the end-users reaction to health applications. The model contains three concepts: 1) the perceived ease of use; 2) the perceived usefulness; and 3) the determination of the intention (intention to use), which influences the actual use [11]. Perceived ease of use means “the degree of ease, associated with the use of the applications”. Perceived usefulness can be defined as “the degree in which an individual believes that using the applications will help him to gain or increase personal performance”.

Although the TAM is mostly used to evaluate information technology, we chose the TAM to examine the face-to-face version as well, in order to compare both versions of the program.

The concepts were measured by using online questionnaires for each program (TAM online and TAM live). Both questionnaires contained seven items concerning usefulness and user acceptance. Due to the different forms (face-to-face and online), the concept perceived ease of use differs in this questionnaire. All questions were answered on a categorical scale with room for additional comments. In addition, there was one question on how much each subject appreciated the program on a scale of 0 to 10 (the higher the score, the more appreciated). Concepts and questions of the TAM are described in Table 2.

**Table 2:** Concepts and questions of the technology acceptance model (TAM)

Perceived usefulness	- Carry out treatment
	- Dealing with the disease in daily life
	- Additional to health care
	- Time investment (worth it)
Perceived user acceptance	- Recommend it to others
	- Participate again (knowing content and form)
Perceived ease of use	- Experienced problems
	- Easy to use
	- Easy to understand
	- Look and feel of the program

#### Peer leaders

The peer leaders were asked to fill in an online TAM-trainer questionnaire to give their opinion on perceived usefulness, perceived ease of use and user acceptance of the program in which they participated. In addition, three questions were asked on fulfilling the role of peer leader (preparation and execution) as well as willingness to lead the training again. The peer leaders were also asked to rate their opinion of the program.

### Adherence and interaction

Adherence to the program was measured after completing the programs by describing how many people had completed the whole course. Also each participant’s presence during the chats on the discussion board and finishing the exercises of the online program were measured. Content of the interaction on discussion board and chat were available in text and were studied after finishing the online program.

### Evaluation of the goals

Participants of both programs were also asked to describe and to evaluate the goals they wanted to achieve within the program. The fulfilling of the goal was evaluated by the participants themselves and written down by the peer leaders. In the face-to-face version of the program, the goals were evaluated verbally during the last session. In the online training goals were evaluated in the last chat session. The young adults were asked to score their satisfaction on achieving their goals on a scale of ‘0’ to ‘10’ (the higher the score, the more satisfied).

### Data analyses

All quantitative data were entered into an SPSS 15.0 database and processed using descriptive statistics. The five possible answers on the concepts ‘perceived usefulness’ were combined in three answers: ‘agree’, ‘agree/disagree’ and ‘disagree’ and analyzed, using frequency scores. An item was considered as sufficient if 2/3 of the participants agreed with the item. Participation in the e-Health application, chat, discussion board, home exercises, was counted throughout the content management system of the website. Additional comments of the participants were collected afterwards by the researcher (JA) and were thematically organized and reported.

Explorative qualitative analyses of the interaction on the e-Health applications were performed by three researchers (JA, LS and HvO). They coded all transcripts independently. Relevant fragments were first categorized into concepts of “topics of discussion”, “type of questions” and content and subsequently categorized into subthemes, using inductive analysis until consensus was reached.

For evaluation of the goals and appreciation of the program, mean and range were computed.

## Results

### Characteristics of the study population

The online self-management program began in January 2011 with two groups of six participants. Eleven of the participants followed the whole program and participated in the study. One dropped out, due to an exacerbation of illness. One participant was lost to follow-up. The face-to-face version started in March 2011. There were no dropouts during the weekend experience though one participant was lost to follow up.

Sixteen participants were female; only 3 males participated in this study. The mean age was 21 years in the face-to-face group and 22 years in the online group. The demographics are described in Table 3.

**Table 3:** Characteristics of the study population

	**Face to face group (n = 9)**	**Online group (n = 10)**
Age, mean (range)	20,7 (17–25)	22,3 (17–25)
Female sex (n)	7	9
Education (n)		
Lower^1^	-	1
Middle^2^	6	7
High^3^	3	2
Diseases		
JIA^4^	3	5
FM^5^	2	2
RA^6^	2	2
SpA^7^	1	1
Immune disease (e.g. MCTD^8^, SLE^9^)	1	-
Duration of the disease, years (SD)	6,4 ± 6,2	5 ± 3,7
Treated by pediatrician or rheumatologist (n)	9	10

^1^Vocational training, ^2^Advanced vocational training, ^3^College/University training, ^4^Juvenile Idiopathic Arthritis, ^5^Fibromyalgia, ^6^Rheumatoid Arthritis, ^7^Spondyloathropathy, ^8^Mixed Connected Tissue Disease, ^9^Systemic Lupus Erythematosus.

All participants of the online program used the Internet on a daily basis. Email, chat and visiting social media were used most often. They all regularly searched for health information on different websites.

### Perceived usefulness and user acceptance of online and face-to-face program

#### O*nline program*

Seven out of ten participants rated the program as useful considering ‘dealing with problems in daily life’. Now that they know the program and form, they would all recommend the program to others and even participate again. Nine out of ten participants indicated the time investment as worthwhile (Table 4).

**Table 4:** Feasibility results: perceived usefulness online en face-to-face program (participants)

	**Online program (n = 10)**			**Face-to-face program (n = 9)**		
**Perceived usefulness**	Agree	Neutral	Disagree	Agree	Neutral	Disagree
Carry out treatment, n	1	5	4	7	2	-
Daily live, n	7	3	-	7	2	-
Additional healthcare, n	6	4	-	7	2	-
Time investment, n	9	1	-	9	-	-
**Perceived user acceptance**						
Recommend the program to others	10			9		
Would participate again	10			6		
Overall score (mean, range)	7.3 (5–10)			7.7 (6–10)		

#### Face-to-face program

The participants of the face-to-face program valued the program as equally useful when considering ‘carry out treatment, daily live and addition to healthcare’. All participants indicated the time investment as ‘worthwhile’. Six out of ten would participate again (Table 4).

### Perceived ease of use

#### Online program

All participants found the online program ‘easy to use’ and appreciated the ‘look and feel’ of the website. There were no great difficulties with logging in or technical bugs and they could easily find what they were looking for.

#### Face-to-face program

The perceived ease of use of the content of the face-to-face program was judged positively by the participants. Relations & intimacy and ‘having control over your life and arthritis’ received the highest scores (Table 5). Positive statements were shared such as ‘very helpful tips and tricks’, ‘love the mood boards’, ‘nice video presentations’ and ‘such a difficult subject like sexuality was also discussed’. Still, there was also some criticism such as: ‘the lack of outdoor activities during the weekend’, ‘healthy nutrition–as sub-theme of sports and exercise–was only generally discussed’, ‘absence of evaluating the goals’ and ‘the need for more theoretical interpretation by the peer leaders’ were noted.

**Table 5:** Feasibility results: perceived ease of use of the face-to-face program

**Perceived ease of use (n = 9)**	**Good**	**Insufficient**
1. Are you a self-manager?	8	1
2. Communication	8	1
3. Feeling blue	8	1
4. Sport and exercise	8	1
5. Relations and intimacy	9	-
6. Having control over your live and arthritis	9	-

### Adherence with the face-to-face and online program

There were no dropouts in the face-to-face weekend program. Twelve participants started the online program; eleven finished it after six weeks. One participant dropped out after three weeks due to an increase of disease activity. Nearly all online participants logged in twice a week on the website and participated in the 90 minutes weekly chat. Two participants missed one chat, due to hospitalization and school activities. Ninety-five percent of the home exercises were finished. In total, 191 messages were posted on the discussion board; 17 messages were sent by the trainer, 174 messages were sent by the participants with a mean number of 21 messages per participant.

### Interaction on the discussion board and chat in the online program

On the discussion board, the main topic of the posted messages was sharing experiences on the themes of the program (for example: what does arthritis mean to you or how do you handle problems) and discussion of questions about the home exercises. Real life examples were given to clarify their experiences. Tips and tricks about dealing with fatigue and dealing with friends were shared. Support was given in case of concerns about their future or communication problems with the health care provider or the mentor at school or work.

Furthermore, individual goals to achieve in the program and future goals were discussed. Feedback and support were given if they had undertaken a step to achieve their goal (for example: talk to the mentor at school). In the group chat, the homework was discussed and the new theme was explained. Despite the fact that they couldn’t see each other–no webcams were used, the young adults shared a lot of personal experiences (for example about being angry and upset and about intimacy and sexuality). Encouragement, tips, tricks and support were also given in this case.

### Goals and achieving goals

The 10 participants of the online training formulated a total of fourteen personal goals; the 9 face-to-face participants formulated a total of fifteen goals (Table 6). After six weeks, for the online training, the young adults were asked to evaluate the achievement of their goals with a number between ‘0’ and ‘10’ (the higher the number, the more satisfied). The mean score was 8.4 (range 6–10). In the face-to-face training, the personal goals were verbally evaluated during the last theme portion of the weekend. Although goals were achieved, the general comment was that this evaluation deserved more attention and time at the end of this weekend.

**Table 6:** Number and summary personal goals, formulated at the start of the online and face-to-face program

Personal goals*	**Online**	**Face-to-face**
Communication about the personal condition with family, friends, school, work	3	2
Handling tiredness, pain and feeling blue	2	3
Carrying responsibility in the personal treatment	1	-
Learn from and modeling with others who have the same problems	1	-
(Maintain) sports and exercises	2	1
Listing to the signals of the body	1	1
Getting support of others	2	1
Setting boundaries (regarding friends, activities)	1	2
Coping with the frustrations of having a chronic diseases	1	2
Getting to know other youngsters, share experiences and problems	-	1
Anger management	-	1
Feeling an outcast; handling and sharing tips and tricks	-	1

*Participants could formulate > one goal.

### Feasibility as judged by peer leaders

#### Online program

Concerning the perceived usefulness, three of the four peer leaders of the online training rated the domain ‘dealing with problems in daily live’ as the most useful. All four peer leaders found the program and the online applications ‘easy to use’ and ‘clear in their purpose’. There were no big technical problems or bugs. All peer leaders were willing to lead the program again. Two peer leaders were surprised by the level of intimacy and frankness the group reached despite the fact that they could not ‘actually see each other live’. Additional comments were made about the preparation needed (‘I didn’t plan enough time to prepare myself’) and the execution of the program (‘is there an online tool available for keeping track of the exercises of the participants?’). The collaboration with the peer leaders was highly appreciated. The program in general received a mean overall score of 7.5 (range 7–8).

#### Face-to-face program

All peer leaders rated that the face-to-face program ‘as useful’ on all aspects. Overall scores on content and form for the face-to-face self-management program were good, except for ‘healthy nutrition’ (a topic of the Sport and Exercise theme). They would recommend the program to others and felt confident ‘to fulfill their role as peer leader’. They were also willing to give the training again. Three out of four peer leaders recommended that ‘there should be more physical activities at the start of the program’. Peer leaders also noticed that more attention should be paid on the theme of feeling blue or depression. The face-to- face program received a mean score of 7.7 (range 6–10).

## Discussion

This study assessed the feasibility of an online and a face-to-face version of a recently developed self-management program ‘Challenge your arthritis’. This program was designed to help young adults with rheumatic diseases manage their chronic health condition. Considering the three domains of feasibility, the young adults and peer leaders appreciated the online and face-to-face program both as useful and found the time investment ‘worthwhile’.

They all would recommend the program in which they participated to other young adults with a rheumatic disease and all stated that they were likely to participate again, now they know the program’s content and form.

To predict the acceptance of a (e-Health) program, we used the TAM model [12] in order to test not only the technique but also the adherence to and acceptance of the program. Although many Health prediction models on the actual use have been developed over the last years, the TAM is one of the most utilized models to underline the positive relationship between the ease of use, perceived usefulness, and the actual use of an intervention.

From the start of the process of development, we chose to have an active involvement with the young adults themselves which could have attributed to the positive outcomes of feasibility. Active involvement of end users is recognized as a critical factor for the actual use of the intervention, by Van Gemert-Pijnen et al. [13]. They strongly emphasize the importance of a holistic framework for the development of an e-Health application in which a co-creation of technology, patients, caregivers but also an evaluation is included [13].

The positive outcomes of the patients and peer leaders on the feasibility of the program may have been influenced by the active involvement of the young adults themselves.

Adherence to both versions of the program was high, the dropout rate was low. This is in contrast with the results of the ‘Eysenbach’ study [14] in which was described that particular self-help applications are known for their high number of dropouts (as being a natural and typical feature). The positive outcomes of this study may be declared by the length of the online program (limited) and ‘the free choice of time to follow the program’ (in their own time, a weekly chat session in the evening) which are known factors to enhance the adherence to an e-Health intervention [15,16].

Stinson et al. [16] explored that young adults have a strong believe that web based programs are a promising avenue for the accessibility and availability for improving self-management behavior. We do not know which factors influenced the individual choice for the face-to face or the online program in our study. Also, we did not examine whether disease duration or nature (subtype) of the rheumatic disease influenced the decision. Future research is needed to explore these aspects.

Based on our own practice with young adults and the theory of Bandura [10], we know young adults ‘learn the most’ of their peers and modeling is an important factor in stimulating positive self-management. Therefore we educated young adults with rheumatic disease to give the training. Although there are other self-management programs for adults with a chronic disease in which peer leaders have a leading role [17-20], little is known about the effects of this concept for young adults with a rheumatic disease. In Stinson’s [21] study on the feasibility of an online self-management training for young adults with a rheumatic disease, a health care professional conducted this specific role, but experiences were not described.

Our study showed that young adults were positive about the content and felt comfortable to talk about personal information in the online program. These findings were also recognized in the feasibility study of White [22] among a comparable group of young adults. The anonymity could be an influencing, positive factor of the online program.

## Conclusions

We have shown that both versions of the self-management program ‘Challenge your arthritis’ are feasible and are appreciated by young adults with a rheumatic disease. The involvement of the end user in the development of the program has contributed to the usefulness, ease of use and user acceptance. The support of self-management of chronically ill young patients is an important task of all health care professionals [2,4,5,8,22]. The face-to-face and online program may prove to be a practical aid to adolescent health practice. We believe a randomized controlled study is needed to investigate these effects.

## Abbreviations

JIA: Juvenile idiopathic arthritis; DRPL: Dutch rheumatism patient league; TAM: Technology acceptance model; MCTD: Mixed connected tissue disease.

## Competing interests

The authors declare that they have no competing interests.

## Authors’ contributions

JA designed and coordinated the study, contributed to data collection, data analyses and interpretation, compiled the manuscript first and final manuscript draft. HvO and AAK were involved in the study design, data analyses and interpretation and drafting the final manuscript. LS and MZ participated in the design of the study, data collected, data analysis, interpretation and compiled the first manuscript draft. AdV, as patient representative of the DRPL and co project leader of this (research) project, helped to coordinate this study. HB endorsed the study and was involved in drafting the manuscript. All authors read and commented on the manuscript. All authors read and approved the final manuscript.
